# Resilience Among Parents of Adolescents With Type 1 Diabetes: Associated With Fewer Parental Depressive Symptoms and Better Pediatric Glycemic Control

**DOI:** 10.3389/fpsyt.2022.834398

**Published:** 2022-04-13

**Authors:** Dan Luo, Yubing Wang, Xue Cai, Ruxue Li, Mingzi Li, Haiyan Liu, Jingjing Xu

**Affiliations:** ^1^School of Nursing, Nanjing University of Chinese Medicine, Jiangsu, China; ^2^Department of Endocrinology, Children's Hospital Affiliated to Nanjing Medical University, Jiangsu, China; ^3^School of Nursing, Peking University, Beijing, China; ^4^Department of Neurology, The Second Affiliated Hospital of Xuzhou Medical University, Jiangsu, China; ^5^Department of Endocrinology, The First Affiliated Hospital With Nanjing Medical University (Jiangsu Province Hospital), Jiangsu, China

**Keywords:** resilience, depressive symptoms, diabetes distress, parents, diabetes mellitus Type 1

## Abstract

**Background:**

Although pediatric resilience plays a significant role in resisting negative moods and improving glycaemic control, little research exists regarding resilience among the parents of adolescents with Type 1 diabetes.

**Objective:**

To investigate parental resilience's correlations with parental depressive symptoms, parental diabetes distress, and pediatric glycaemic control.

**Methods:**

This cross-sectional study recruited adolescents with Type 1 diabetes and their parents from two hospitals. The parents completed questionnaires. The 10-item Connor-Davidson Resilience Scale measured resilience; the Problem Areas in Diabetes Survey-Parent Revised version measured diabetes distress; the Patient Health Questionnaire-9 measured depressive symptoms. Standard glycated hemoglobin tests were performed on the adolescents.

**Results:**

Data from 224 parents (77.2% female, M_age_ = 39.88 [SD = 5.02], age range = 30–56 years) of adolescents (50.9% boys, M_age_ = 13.54 years [SD = 2.48], age range = 10–19 years) were available. More than half (52.7%) of parents exceeded the criterion score for high resilience. Parental resilience was significantly negatively associated with parental depressive symptoms and diabetes distress. Parents from the high-resilience group reported fewer depressive symptoms than those from the low-resilience group. In multivariate regressions, greater parental resilience is consistently related to better pediatric glycaemic control beyond parental psychological risk factors.

**Conclusions:**

This study highlights the importance of parental resilience for parental mental health and glycaemic control among adolescents with Type 1 diabetes. The appropriate resilience support programme might be developed for parents, especially for those existing depressive symptoms and diabetes distress.

## Introduction

Type 1 diabetes is one of the most common childhood diseases which requires lifelong insulin treatment (1). The incidence of Type 1 diabetes has been increasing worldwide, especially among children under 19 (2). Deteriorating glycemic control is shared among adolescences with Type 1 diabetes due to hormonal changes and problematic self-management behavior (3). The pivotal Diabetes Control and Complications Trial demonstrated that elevated glycosylated hemoglobin (HbA1c) was associated with long-term complications, impaired neurocognitive function, and increased mortality (4, 5).

Parents of adolescents with Type 1 diabetes are responsible for the complex management of diabetes, leading to caregiver burden and stress (6). Moreover, they bear a heavy financial burden, experience frequent family conflict, and fear the complications of diabetes (7–9). In a systematic review, 19% of parents experienced psychological distress lasting 1–4 years after their children were diagnosed with Type 1 diabetes (10). Bassi (11) reported that the prevalence of depression among the parents of adolescents with Type 1 diabetes ranged from 13 to 74% in different studies. Evidence from empirical research suggests that parental depressive symptoms and diabetes distress are negatively associated with pediatric glycaemic control (12, 13). Identifying protective factors that could play dual roles to relieve parents' depressive symptoms and diabetes distress and improve glycaemic control among adolescents is crucial to improving the quality of life for these families.

One factor that has attracted considerable attention in pediatric chronic diseases is caregiver resilience, typically defined as an individual's capacity to resist adverse psychological reactions and demonstrate positive outcomes when caring for a child with chronic illness (14). Among parents of children with cancer, those in the high-resilience group displayed fewer depressive symptoms and reported lower levels of uncertainty regarding the illness than those in the low-resilience group (15). Similarly, Rodríguez-Rey et al. (16) conducted a longitudinal study. They found that parental resilience was a strong negative predictor of anxiety, depression, and posttraumatic stress disorder following their child's treatment in intensive care. On the other hand, parental resilience has been linked to the health outcomes of children in recent studies. Khu et al. (17) reported that parental resilience was positively associated with pediatric pain indicators among adolescents diagnosed with chronic pain. In contrast, Gmuca et al. (18) argued that no significant correlation existed between parental resilience and adolescents' pain levels.

According to the pediatric transactional theory (19), the health status of children is deeply affected by the bidirectional interactions that occur between parents and children. Most type 1 diabetes studies have focused primarily on adolescents' resilience and proved that greater resilience was associated with better glycemic control and quality of life (20, 21). Only one survey has specifically examined the association between parental resilience and depressive symptoms. Edraki and Rambod (22) reported that parents in the lowest resilience group experienced extremely severe stress and depression. No study has explored the association between parental resilience and pediatric glycaemic control.

In summary, glycaemic control is suboptimal among adolescents with Type 1 diabetes. There is a well-documented association between parents' negative emotions and the glycaemic control of the children. Therefore, identifying the key variables that correlate with both negative parental emotions and pediatric glycaemic control may provide a rational basis for developing effective interventions. This study hypothesized that higher parental resilience would be associated with fewer parental depressive symptoms, lower parental diabetes distress, and better pediatric glycaemic control.

## Materials and Methods

### Participants and Procedure

This cross-sectional survey study was conducted from February 2020 to July 2021. Participants were consecutively recruited from two academic hospitals in China. The eligibility requirements included: being the parents of adolescents (aged 10–19 years) who were diagnosed with Type 1 diabetes for over 6 months; responsible for adolescents who receive either multiple daily injections or continuous subcutaneous insulin infusions; being mentally and physically competent to answer the study questionnaires; able to read and speak Chinese; being willing to participate in the study. Parents were excluded if they had any psychiatric disorders or comorbid organic diseases or if their children were taking any medications that could influence glycaemic control (such as glucocorticoid drugs). The study sample size was calculated based on a pilot study. Among the 20 parents of adolescents with Type 1 diabetes in the pilot study, Pearson's correlation coefficients between parental resilience and depressive symptoms, diabetes distress, and adolescents' HbA1c value were −0.38, −0.31, −0.23, respectively. Therefore, based on α = 0.05, β = 0.90, and *r* = 0.23, a 195-subject sample size was estimated to be necessary (23).

**Figure 1:**
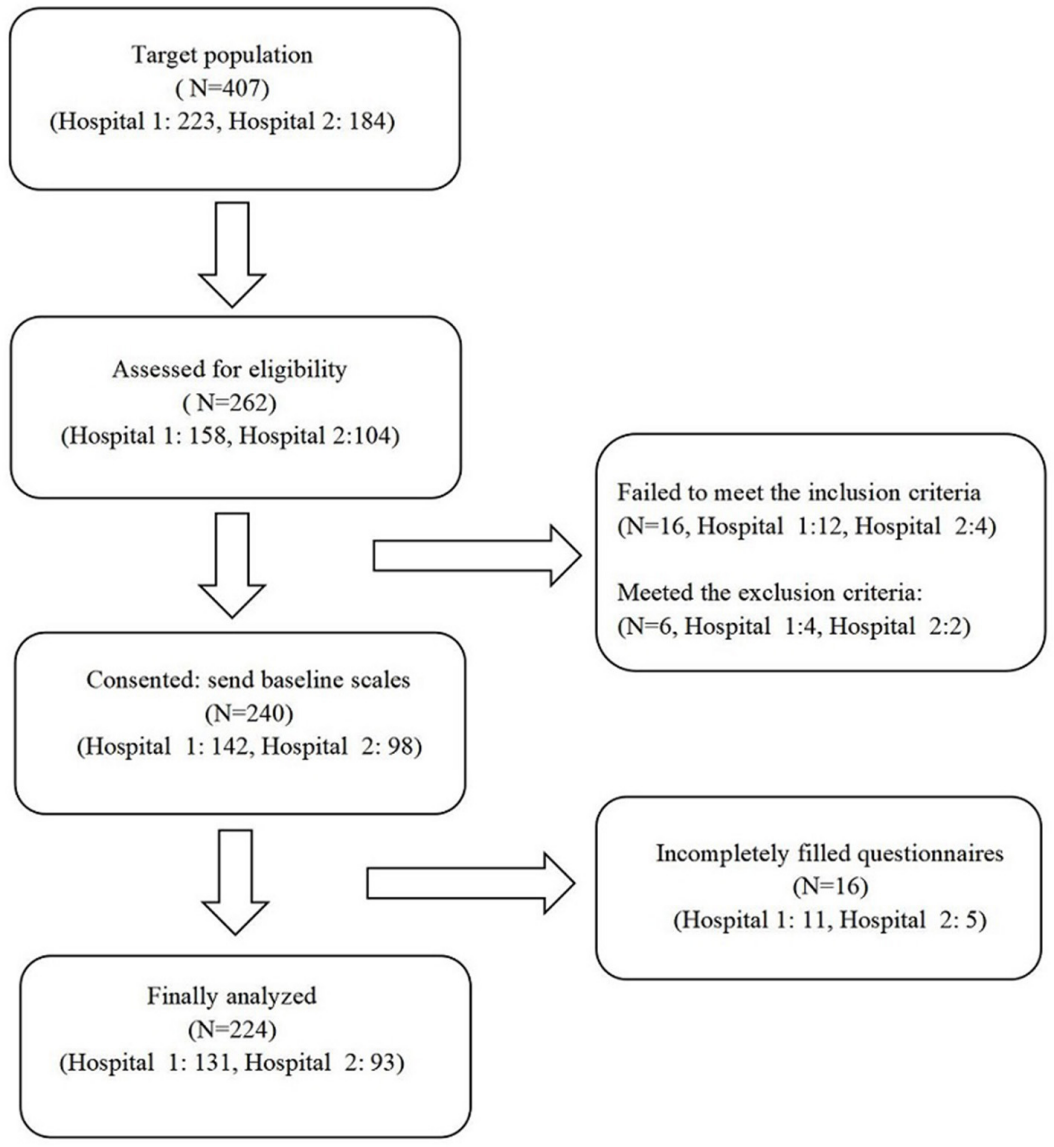
Participant recruitment flow chart.

A total of 407 adolescents with Type 1 diabetes visited the endocrine clinic of these hospitals during the study period. All parents were contacted by a diabetes specialist nurse and asked if they were interested in participating in the study. Of the 262 parents interested in participating and screened for eligibility, 240 were eligible. Eligible parents signed the informed consent and finished the questionnaires. Because of the incompletely filled questionnaires, 16 respondents were excluded from the study. Finally, the data of 224 participants were used for the data analysis. Figure 1 shows the participant recruitment process in detail.

### Data Collection

The parents who signed the informed consent form completed the pen-and-paper survey in a quiet room. The self-reported questionnaires included five parts: parental resilience, depressive symptoms, diabetes distress, demographic characteristics, and adolescents' demographics and disease information. Well-trained diabetes education nurses instructed the parents to respond to the questionnaires. The entire survey took ~20 min to complete, and all questionnaires were reviewed on-site to improve quality and completeness. While the parents filled out the questionnaires, the adolescents underwent HbA1c testing.

### Instruments

#### Demographics

The self-designed questionnaire used in this study consisted of two separate sections. The first section asked about parent demographic characteristics (age, gender, education level, marital status, family income, work conditions, the number of children, and whether the parent is the only caregiver). The second section included questions regarding the demographic and disease-related information of the adolescents with Type 1 diabetes (age, gender, duration of Type 1 diabetes, insulin therapy method, whether they monitor blood glucose daily, and self-management level).

#### Resilience

The Chinese version of the 10-item Connor-Davidson Resilience Scale (CD-RISC-10) was used to assess parental resilience (24). The CD-RISC-10 is a 5-point Likert scale (0: never to 4: almost always) that measures the personal capacity to tolerate difficulty and achieve positive outcomes. All item scores were summed to obtain a total score, with higher scores reflecting greater resilience. The scores of the Chinese version of the CD-RISC-10 can also be divided into two levels: 0–25 indicates low resilience, whereas 26–40 indicates high resilience. The Chinese version of the CD-RISC-10 has well-documented reliability (Cronbach's α = 0.88, the 2 week test-retest = 0.73) (24).

#### Depressive Symptoms

The depressive symptoms of parents were evaluated using the Chinese version of the Patient Health Questionnaire-9 (PHQ-9) (25). The PHQ9 is not a diagnostic instrument but does give an indication of the severity of depressive symptoms experienced. A four-point scale (0: not at all to 3: nearly every day) was used for all nine items. The total scores ranged from 0 to 27, with higher scores indicating more depressive symptoms. Parents with a PHQ-9 score of 10 or higher are considered to be suffering from severe depression symptoms. They are offered assistance to obtain further assessment and treatment. The Cronbach's α for the Chinese version of the PHQ-9 was 0.86, and the 2-week test-retest was also 0.86.

#### Diabetes Distress

To measure the diabetes distress of parents, we used the Problem Areas in Diabetes Survey-Parent Revised version (PAID-PR), which was designed by Markowitz et al. (26). Two subscales for immediate distress and theoretical distress were examined, each consisting of nine items. The 18-item PAID-PR utilizes a 6-point Likert scale (1: no concern to 6: serious concern), with total scores ranging from 18 to 108, and higher scores indicate more diabetes distress. We translated the original PAID-PR into Chinese through the following four stages: (1) forward translation, (2) back translation, (3) committee discussion, and (4) pilot test. The Chinese version of PAID-PR in this study demonstrated good construct validity (χ^2^/DF = 2.29; CFI = 0.90; RMSEA = 0.08) and reliability (Cronbach's α = 0.91).

#### Glycaemic Control

Capillary blood samples were collected from adolescents through finger sticks. HbA1c values were measured at a central laboratory shared by both recruitment sites utilizing the standard method (Clover A1c Analyzer, Bio-Rad D10 hemoglobin testing system Specifications).

#### Ethical Considerations

The Bioethics Committee of the Peking University Health Science Center approved this study (IRB00001052-19108). All study procedures were following the Declaration of Helsinki (World Medical Association, 2013).

### Statistical Analyses

Statistical analyses werfgv5©^1^e conducted using SPSS 22.0 (IBM Corporation, New York, NY, USA). The study population is described by mean (Standard deviation, SD) and *n* (%). The Pearson correlation coefficients (*r*) was calculated to evaluate the associations among parental resilience and depressive symptoms and diabetes distress. The difference between *r* for resilience-depressive symptoms and *r* for resilience-diabetes distress were explored following the method provided by Lee et al. (27). The odds ratios (ORs) and 95% confidence intervals (CIs) for risk of low resilience (CD-RISC-10 score ≤ 25) in relation to every 1-SD higher of depressive symptoms and diabetes distress were obtained using logistic regression analyses. The subgroup analysis was employed to evaluate the impact of parental gender on the associations that depressive symptoms and diabetes distress had with the risk of low resilience. We established three linear regression models to evaluate the independent association between parental resilience and pediatric glycemic control. We centered all predictor variables for testing two-way interactions. The collinearity test was performed using the Variance Inflation Factor (VIF) and Tolerance (TOI), and the results showed no overlapping. Model 1 was adjusted for variables including child age, gender, disease duration, insulin regimen, daily blood glucose monitoring, and self-management level that demonstrated a correlation with pediatric glycaemic control in previous studies (28, 29). For example, insulin pump therapy, higher adherence for blood glucose monitoring, and better self-management were associated with lower HbA1c (30). Model 2 was adjusted for variables in Model 1 plus parental depressive symptoms and diabetes distress. Model 3 was adjusted for variables in Model 2 plus interaction effects between parental resilience and depressive symptoms and diabetes distress. For the unranked variables, dummy variables were created. For all anal yes, *p* < 0.05 indicated significance.

## Results

### The Characteristics of the Participants

The mean age of the parents was 39.88 (SD = 5.02) and ranged from 30 to 56 years. Most respondents were mothers (77.2%), and almost all (95.0%) of the parents reported being married. In addition, 31.2% of the parents were unemployed, and 52.2% had a family monthly income of <5,000 Yuan. Over half (53.1%) of the parents reported having more than one child. Additionally, 21.0% of parents care for adolescents alone. Among the adolescents with Type 1 diabetes, 49.1% were boys, and 61.6% were younger than 15 (ranged from 10 to 19), with a mean age of 13.54 (SD = 2.48) years. Their average Type 1 diabetes duration was 3.91 (SD = 2.77) years, and 76.3% were currently using an insulin pen for insulin injection. Parents reported that 88.4% of adolescents monitored their blood glucose levels daily, and 30% had low levels of self-management. The mean HbA1c was 8.0 ± 1.8%, and 52.7% of adolescents did not reach the glycaemic goal (HbA1c <7.5%) (31). See detail in Table 1.

**Table 1:** Participants' characteristics.

**Participants**	**Characteristic**	**Classification**	**Number (n)**	**Percentage (%)**
Parents	Gender	Female	173	77.2
		Male	51	22.8
	Age(years)^†^	<40	108	48.2
		≥40	116	51.8
	Education level	Primary education	17	7.6
		Secondary education	134	59.8
		Higher education	73	32.6
	Marital status	Married	212	95.0
		Divorced	12	5.0
	Work status	Employed	154	68.8
		Unemployed	70	31.2
	Family monthly income	<5,000 Yuan	117	52.2
		≥5,000 Yuan	107	47.8
	Number of children	1	105	46.9
		≥ 2	119	53.1
	Sole caregiver	Yes	177	79.0
		No	47	21.0
Adolescents	Gender	Girl	114	50.9
		Boy	110	49.1
	Age(years)^‡^	<15	138	61.6
		≥15	86	38.4
	Diabetes duration(years)^§^	<5	164	73.2
		≥5	60	26.8
	Insulin regimen	Pen	171	76.3
		pump	53	23.7
	Daily blood glucose monitoring	Yes	198	88.4
		No	26	11.6
	Self-management level	Low	60	26.8
		Moderate	156	69.6
		High	8	3.6
	HbA1c, % ^¶^	≤ 7.5	106	47.3
		>7.5	118	52.7

*SD, Standard deviation; HbA1c, Glycated hemoglobin*;

† *The mean (SD) for parents' age was 39.88 (5.02) years*;

‡ *The mean (SD) for adolescents' age was 13.54 (2.48) years*;

§ *The mean (SD) for diabetes duration was 3.91 (2.77) years*;

¶ *The mean (SD) for HbA1c was 8.0 (1.8)%*.

### Resilience, Depressive Symptoms and Diabetes Distress Among Parents

The Cronbach's α of CD-RISC-10, PHQ-9, and PAID-PR in this sample were 0.88, 0.88, and, 0.91, respectively, indicating excellent reliability. Just over half of parents (52.7%) were highly resilient with a CD-RISC-10 score above 26, and a mean score was 28.36 (SD = 6.81); 12.9% experienced severe depression symptoms, PHQ-9 score ≥ 10, and the mean score was 4.67 (SD = 4.49). The means score for diabetes distress was 65.68 (SD = 19.82). Regarding gender differences, mothers reported more depressive symptoms than fathers (*t* = −2.12, *p* = 0.032). No significant differences were found between mothers and fathers regarding resilience and diabetes distress.

The results of Pearson correlation analysis were shown in Table 2, parental resilience was moderately negatively associated with parental depressive symptoms (*r* = −0.43, *p* < 0.001) and diabetes distress (*r* = −0.37, *p* < 0.001). There was no statistical difference between the *r*_1_ (resilience and depressive symptoms) and *r*_2_ (resilience and diabetes distress) (*z* = −1.10, 95%CI: −0.21, - 0.06).

**Table 2:** Levels and associations of parental resilience with parental depressive symptoms and diabetes distress.

	**Mean**	**SD**	**Correlation Matrix**		
			**1**	**2**	**3**
Resilience	28.36	6.81	1		
Depressive symptoms	4.67	4.49	−0.43**	1	
Diabetes distress	65.68	19.82	−0.37^**^	0.53**	1

** *Correlation is significant at the 0.01 level (2-tailed)*.

Following the bivariate analysis, we included parental depressive symptoms and diabetes distress in a logistic regression model, in which the dichotomous dependent variable was parental resilience level (low resilience: CD-RISC-10 score ≤ 25). More parental depressive symptoms were associated with an OR of 1.15 (95% CI: 1.06–1.25, *p* = 0.001) for the low level of resilience. The prevalence of low resilience was significantly higher in parents who had severer diabetes distress (OR = 1.02, 95%CI: 1.01–1.04, *p* = 0.036). Subgroup analysis showed that the association between depressive symptom and resilience was significant in both mothers (OR = 1.11, 95%CI: 1.02–1.21, *p* = 0.019) and fathers (OR = 1.40, 95%CI: 1.08–1.82, *p* = 0.012). Furthermore, diabetes distress did not lead to an increased risk of low resilience in fathers (*p* = 0.902), while did in mothers (OR = 1.02, 95%CI: 1.00–1.04, *p* = 0.032).

### Multivariate Analysis of the Association Between Parental Resilience and Pediatric Glycemic Control

Three linear regression models were established to evaluate the association between parental resilience and pediatric glycaemic control (Table 3). The model 1 showed that greater parental resilience was correlated with lower pediatric HbA1c after controlling for adolescents' demographic and disease characteristics (β = −0.06, *p* = 0.002, Cohen's *d* = 0.44). The same association was observed in model 2, adjusted for variables in Model 1 plus parental depressive symptoms and diabetes distress (β = −0.05, *p* = 0.008, Cohen's d = 0.37). Two-way interactions between parental resilience and depressive symptoms and diabetes distress were added in model 3, and results found no significant interactions. The association between parental resilience and pediatric glycemic control remained unchanged (β = −0.05, *p* = 0.007, Cohen's d = 0.38).

**Table 3:** Regression analyses testing parent resilience as predictor of HbA1c.

**Variables**	**Model 1^†^**		**Model 2^‡^**		**Model 3^§^**	
	**β**	***p***	**β**	***p***	**β**	***p***
Child gender	0.44	0.059	0.40	0.082	0.42	0.075
Child age	0.04	0.475	0.02	0.694	0.02	0.642
Disease duration	0.02	0.693	<0.00	0.923	<0.00	0.945
Insulin regimen	−0.32	0.261	−0.25	0.375	−0.29	0.318
Daily blood glucose monitoring	−0.60	0.117	−0.63	0.092	−0.64	0.089
Self-management level (1)	−0.07	0.792	−0.05	0.860	−0.03	0.911
Self-management level (2)	−1.29	0.052	−1.17	0.077	−1.27	0.062
Resilience	−0.06	0.002	−0.05	0.008	−0.05	0.007
Diabetes distress			0.02	0.008	0.02	0.006
Depressive symptoms			−0.05	0.104	−0.06	0.107
Resilience × Diabetes distress					< -0.01	0.625
Resilience × Depressive symptoms					< -0.01	0.816
R2		0.11^*^		0.13^*^		0.13^*^

*HbA1c, glycosylated hemoglobin*;

* *, P <0.01*.

† *Model 1: adjusted for child age, disease duration, insulin regimen(pen or pump), daily blood glucose monitoring (no or yes), and self-management level(low or moderate, low or high)*.

‡ *Model 2: Model 1 plus diabetes distress and depressive symptoms*.

§ *Model 3: Model 2 plus interactions between resilience and depressive symptoms and distress*.

## Discussion

The primary aim of the present study was to explore the correlations that parental resilience has with parental depressive symptoms, parental diabetes distress, and pediatric glycaemic control. Our analysis provided support for our hypotheses. Among parents of adolescents with Type 1 diabetes, higher parental resilience was associated with fewer parental depressive symptoms and lower diabetes distress. Moreover, parental resilience had an independent effect on pediatric glycaemic control after statistically controlling for adolescents' demographic and disease variables and parental depressive symptoms and diabetes distress.

The mean PHQ-9 score of parents in this study was higher than that of a Chinese community population (25). Complex, diabetes-specific daily tasks, and frequent hospital visits increase parents' vulnerability to experiencing depressive symptoms (13). Specifically, mothers of adolescents with Type 1 diabetes reported more depressive symptoms than fathers. Women's susceptibility to depression and a higher level of maternal involvement in diabetes care may account for this difference (32). The mean score of parental diabetes distress in our study was much higher than the results reported for a cohort in the United States (26). The lower incidence of Type 1 diabetes in China may result in specific challenges for parents and adolescents with Type 1 diabetes in this country. In a cross-national survey, 19.1% of Chinese participants reported being discriminated against because of their diabetes, compared with 10.6% of participants in the United States (33). Moreover, Chinese universities and junior colleges are allowed to refuse admission to students with Type 1 diabetes according to government regulations, which may increase parents' uncertainty about their children's futures (34). Another significant finding of our study was the relatively high level of resilience identified among the parents of adolescents with Type 1 diabetes compared to a general Chinese population (35). Resilience theory emphasizes that stressful circumstances will provide opportunities to improve resilience, allowing the individual to maintain their physical and psychological well-being (36). From a cultural perspective, Chinese people typically regard suffering and hardships as necessary conditions for growth and success. They attempt to stay positive and pay less attention to unwelcome thoughts.

In addition, we found that parental resilience was negatively correlated with parental diabetes distress and depressive symptoms. This finding was similar to the findings of the studies conducted by Tully (37) and Ye et al. (38), who surveyed the parents of children with asthma and cancer, respectively. Mason et al. (39) conducted a longitudinal qualitative study examining the mothers of children with an autism spectrum disorder and reported that higher baseline resilience among mothers predicted lower stress trajectories over 18 months. The neuro mechanism of resilience could explain these results. An increasing body of evidence suggests that resilience could invoke specific brain structures and neural circuits to help the individual to regulate mood (40, 41). Although parental psychosocial screening is becoming more common at diabetes clinics, the medical staff is more likely to recognize and relieve negative emotions than evaluate and improve positive psychological qualities (42). Identifying the resilient characteristics of parents can help medical staff to provide specific family-centered education and support.

The current study provides initial evidence that parental resilience was positively correlated with better pediatric glycaemic control among adolescents with Type 1 diabetes. This finding extends previous work regarding the correlation between parental psychological variables and adolescents' diabetes-related outcomes. The mechanisms underlying this positive relationship may be partly explained by pediatric transactional theory (19). The transactional theory highlights the interactions between the parents' characteristics and children's behaviors and health outcomes. Greater resilience can lead to higher self-efficacy and more positive coping strategies when individuals encounter traumatic circumstances (43). Lohan and Mitchell (44) demonstrated that parental self-efficacy was positively associated with adolescents' diabetes self-management behavior. Speculatively, parents with higher resilience may have more confidence in diabetes care, providing more freedom and support to their children, which might sequentially improve self-care behavior and glycaemic control of adolescents (45).

This study has several strengths. First, our results may enable cross-cultural comparisons because our measures are well-established and have good psychometric properties among people from different countries. Second, the HbA1c values were tested using a standard method. Third, we included demographic, disease-related, and psychological covariates in serial multivariable regression models and demonstrated that parental resilience was independently correlated with pediatric glycaemic control. We also acknowledge several limitations of our study. First, the cross-sectional design makes it impossible to determine causal relationships between parental resilience and other variables. More longitudinal studies and randomized controlled trials remain necessary. Second, the study consists of samples from two Chinese hospitals, and our observations could be characteristic of that specific population studied. The reader may need to be cautious in interpreting the results, and more extensive studies are needed to explore whether our conclusions are replicable in different countries with diverse cultures. Third, the sample size of fathers was small, reducing the statistical power to detect differences in resilience and diabetes distress between mothers and fathers. More evenly sized samples and the inclusion of both parents from a family unit might allow a more accurate examination of gender differences. Last, the parents of the presents study were invited on a voluntary and anonym basis. Volunteers usually have a better mental function, and this means that our results could have been biased.

The current study demonstrated that in parents of adolescents with Type 1 diabetes, resilience might be a promising focus for interventions to improve parental mental health and pediatric glycaemic control. As applied to clinical care, we recommend incorporating resilience in the routine assessment of parental psychological symptoms. Standard screening of parents in diabetes clinics may help identify those who need emotional support to address depressive symptoms and diabetes distress and are most likely to benefit from resilience intervention. Some resilience strengthening strategies that medical staff can adopt include discovering and utilizing parental internal strengths (optimistic, self-efficacy and calm) and organizing activities to promote interaction between parents and children (46, 47). In addition, the findings from parents of adolescents with Type 1 diabetes could be generalized to the parents of children with chronic disease. There is an urgent need for research focusing on the relationship between parental positive psychological variables and pediatric disease-related health outcomes.

In conclusion, the parents of adolescents with Type 1 diabetes showed relatively high resilience. Higher parental resilience was associated with fewer parental depressive symptoms and lower levels of diabetes distress. Parental resilience appears to play a significant role in pediatric glycaemic control, adjusting for adolescents' demographic and clinical parameters and parental risk psychological variables. Future research should further explore the effects of parental resilience on parental mental health and pediatric diabetes management and examine whether resilience-focused interventions can improve the health outcomes of parents and adolescents.

## Data Availability Statement

The raw data supporting the conclusions of this article will be made available by the authors, without undue reservation.

## Ethics Statement

The studies involving human participants were reviewed and approved by Ethics Committee of Peking University. Written informed consent to participate in this study was provided by the participants' legal guardian/next of kin.

## Author Contributions

DL designed the study, enrolled participants, analyzed and interpreted the data, and wrote the manuscript. YW, XC, RL, and JX were responsible for collecting data. ML, HL, and JX supervised this research project.

## Conflict of Interest

The authors declare that the research was conducted in the absence of any commercial or financial relationships that could be construed as a potential conflict of interest.

## Publisher's Note

All claims expressed in this article are solely those of the authors and do not necessarily represent those of their affiliated organizations, or those of the publisher, the editors and the reviewers. Any product that may be evaluated in this article, or claim that may be made by its manufacturer, is not guaranteed or endorsed by the publisher.

## Footnotes

^1^0020.
